# Burnout prevalence and contributing factors among healthcare workers during the COVID-19 pandemic: A cross-sectional survey study in an urban community in Thailand

**DOI:** 10.1371/journal.pone.0269421

**Published:** 2022-08-04

**Authors:** Jadsada Kunno, Busaba Supawattanabodee, Chavanant Sumanasrethakul, Budsaba Wiriyasirivaj, Pataraporn Yubonpunt

**Affiliations:** 1 Department of Research and Medical Innovation, Faculty of Medicine Vajira Hospital, Navamindradhiraj University, Bangkok, Thailand; 2 Department of Urban Medicine, Faculty of Medicine Vajira Hospital, Navamindradhiraj University, Bangkok, Thailand; 3 Department of Obstetrics and Gynecology, Faculty of Medicine Vajira Hospital, Navamindradhiraj University, Bangkok, Thailand; 4 Department of Public Health, Faculty of Public and Environmental Health, Huachiew Chalermprakiet University, Samutprakan, Thailand; Cardiac Surgery Unit, Papardo Hospital, ITALY

## Abstract

**Background:**

Burnout is associated with an increased risk for severe COVID-19. Few studies have examined burnout prevalence related to healthcare workers during the pandemic. This study investigated the burnout prevalence and contributing factors among HCWs, including medical staff and support staff, during the COVID-19 pandemic in an urban community in Thailand.

**Methods:**

A cross-sectional online survey was distributed among HCWs in Bangkok, Thailand, from July–August 2021. The independent t-test and one-way analysis of variance (ANOVA) were used to compare the contributing factors and burnout items. Variable factors associated with burnout among HCWs were used in multiple linear regression models.

**Results:**

A total of 517 HCWs’ survey responses were received. Most participants were medical staff (55.3%), female (83.4%), and over the age of 35 (59.4%); most participants (65.6%) did not have any diseases but had family members that did (63.6%). The prevalence of overall burnout presented among medical staff (25.9%). The results of the multiple linear regression models found that female (vs. male, β 0.088; 95% CI 0.033, 6.614) was higher associated with overall burnout score. In addition, hours of sleep as > 6 hr./day (vs. ≤ 6 hr./day, β -0.120; 95% CI -6.012, -0.969) was lower associated with overall burnout score.

**Conclusion:**

This study highlights the importance of addressing burnout among HCWs, in which female medical staff who slept less than six hours per day were associated with burnout. Our study further suggested that both intervention and identification are needed of frontline HCWs to prevent and reduce the risk of burnout, as the proportion of females compared to males is high. Thus, the government should provide support in these areas to prevent a humanitarian crisis.

## Introduction

In December 2019, a cluster of patients with pneumonia of unknown cause was linked to a seafood wholesale market in Wuhan, China [1, 2]. Most people infected with the COVID-19 virus will experience a mild to moderate respiratory illness and recover without requiring special treatment [3]. Many countries have experienced multiple waves of coronavirus outbreaks. During the 2020 pandemic, empirical data show that the characteristics varied between waves [4]. In Thailand, sources of risk factors include close contact with a previously confirmed patient, community risk, cluster communities, and active and community surveillance [5]. From this evidence, risk factors responsible for the increased infection among healthcare workers (HCWs) can mitigate the transmission of COVID-19 among healthcare workers and patients alike [6].

HCWs are at the forefront in the containment of COVID-19 and hence are at an increased risk of exposure to COVID-19 [7]. However, COVID-19 represents an occupational health risk among HCWs due to their frequent exposure to infected individuals and higher risk rate than others in the population [8]. This evidence might lead to some HCWs having burnout during work. Working during the epidemic situation, the personnel of every department require being physically strong, and mental health is very important. HCWs are at high risk of stress, anxiety, and depression, including burnout at work, and this may have long-term psychological implications.

While several studies have outlined the prevalence of burnout, less is known about burnout among HCWs. Burnout can be defined as a syndrome resulting from chronic workplace stress that has not been successfully managed [9]. Burnout is synonymous based on with fatigue, stress, or depression, characterized by energy depletion or emotional exhaustion, negativity related to one’s job, and reduced professional efficacy [10–12]. Burnout can result from increased work demands and decreased job resources, along with value conflicts [13]. From this evident lead to n further magnified during COVID-19 pandemic. At this moment, HCWs might be at risk of burnout during the pandemic, and a lot of research has identified several risk factors at the individual and situational levels.

Burnout prevalence among HCWs during COVID-19 has been increasing according to several studies [14–20]. However, few have focused on the burnout of HCWs in particular. Thus, evidence of HCWs’ burnout during COVID-19 is limited to the Bangkok urban community in Thailand. The present study helps address this gap by conducting a cross-sectional survey study among HCWs in Bangkok, Thailand. This study is specifically aimed at burnout prevalence and contributing factors among HCWs, including medical staff and support staff during the COVID-19 pandemic. This study can help identify various characteristics of HCWs who are more likely to be vulnerable to the effects of COVID-19. In addition, understanding how the virus spreads reinforces the importance of prevention measures. Knowing how COVID-19 has impacted people may reinforce the need for everyone to adopt health-promoting behaviors.

## Methods

### Study design

This study conducted a cross-sectional survey of HCWs working at the Faculty of Medicine at the Vajira Hospital in Bangkok, Thailand. Data collection occurred from July–August 2021. This study was approved by the ethics committee of the Faculty of Medicine, Vajira Hospital, Navamindradhiraj University, Bangkok, Thailand and Faculty of Public and Environmental Health, Huachiew Chalermprakiet University, Samutprakan, Thailand, (COA 116/2564) and (อ.1102/2564 ลว), respectively. (See S1 File for details.)

### Participants

HCWs aged 18 years and above agreed to participate in the study. All participants were of Thai nationality and living in the urban community of Bangkok. The sample size was calculated using G*Power based on the estimated population of HCWs in the city.

### Data collection

The questionnaires were completed using an online survey (Google Forms). Participants were recruited on social media using a snowball technique based on social distancing during the COVID-19 pandemic. The invitation requested voluntary participation of HCWs and provided instructions for filling in the questionnaire.

### Questionnaire

The questionnaire was designed based on a previous burnout prevalence and factors contributing to HCWs and adapted to the situation in Thailand by a team of experts.

The questionnaire takes about 20 minutes to complete and is divided into four sections (see S2 File for details). The first part collected socio-demographic information, gender, age (years), education, occupation, income (baht), marital status, children, diseases, family diseases, residence, work experience (years), days off (day per month), hours slept per day, number of colleagues, and COVID-19 experience (Table 1).

**Table 1:** Demographic characteristics and burnout prevalence among healthcare workers (n = 517).

Socio-demographics			Burnout prevalence			
Variable factors		*n* (%)	Overall prevalence of burnout	Personal-related	Work-related	Client-related
			*n* (%)			
Occupational						
	Medical staff	286 (55.3%)	74(25.9%)	84(29.4%)	75(26.2%)	98(34.3%)
	Support staff	231(44.7%)	43(18.6%)	55(23.8%)	40(17.3%)	63(27.3%)
Gender						
	Male	86(16.6%)	14(16.3%)	19(22.1%)	13(15.1%)	20(23.3%)
	Female	431(83.4%)	103(23.9%)	120(27.8%)	102(23.7%)	141(32.7%)
Age (Years)						
	≤ 35	210(40.6%)	46(21.9%)	54(25.7%)	46(21.9%)	66(31.4%)
	> 35	307(59.4%)	71(23.1%)	85(27.7%)	69(22.5%)	95(30.9%)
Education						
	< bachelor	123(23.8%)	30(24.4%)	36(29.3%)	28(22.8%)	45(36.6%)
	≥ bachelor	394(76.2%)	87(22.1%)	103(26.1%)	87(22.1%)	116(29.4%)
Income (Baht)						
	≤ 25,000	254(49.1%)	54(21.3%)	71(28.0%)	49(19.3%)	82(32.3%)
	> 25,000	263(50.9%)	63(24.0%)	68(25.9%)	66(25.1%)	79(30.0%)
Marital status*						
	Sigle	273(52.8%)	60(22.0%)	73(26.7%)	66(24.2%)	81(29.7%)
	Married	208(40.2%)	49(23.6%)	54(26.0%)	43(20.7%)	71(34.1%)
	Separated	36(7.0%)	8(22.2%)	12(33.3%)	6(16.7%)	9(25.0%)
Children						
	No	302(58.4%)	70(23.2%)	83(27.5%)	76(25.2%)	91(30.1%)
	Yes	215(41.6%)	47(21.9%)	56(26.0%)	39(18.1%)	70(32.6%)
Diseases						
	No	339(65.6%)	72(21.2%)	87(25.7%)	74(21.8%)	107(31.6%)
	Yes	178(34.4%)	45(25.3%)	52(29.2%)	41(23.0%)	54(30.3%)
Family diseases						
	No	188(36.4%)	39(20.7%)	48(25.5%)	34(18.1%)	64(34.0%)
	Yes	329(63.6%)	78(23.7%)	91(27.7%)	81(24.6%)	97(29.5%)
Residence*						
	Home	272(52.6%)	66(24.3%)	80(29.4%)	65(23.9%)	91(33.5%)
	Condominium	180(34.8%)	37(20.6%)	41(22.8%)	32(17.8%)	47(26.1%)
	Hospital	65(12.6%)	14(21.5%)	18(27.7%)	18(27.7%)	23(35.4%)
Work experience (Years)						
	≤ 10	221(42.7%)	50(22.6%)	59(26.7%)	50(22.6%)	68(34.0%)
	> 10	296(57.3%)	67(22.6%)	80(27.0%)	65(22.0%)	93(29.3%)
Days off (per month)						
	< 8	200(38.7%)	56(28.0%)	57(28.5%)	53(26.5%)	68(34.0%)
	≥ 8	317(61.3%)	61(19.2%)	82(25.9%)	62(19.6%)	93(29.3%)
Sleep (hours per day)						
	≤ 6	318(61.5%)	81(25.5%)	94(29.6%)	79(24.8%)	108(34.0%)
	> 6	199(38.5%)	36(18.1%)	45(22.6%)	36(18.1%)	53(26.6%)
Number of colleagues						
	≤ 3 persons	430(83.2%)	97(22.6%)	114(26.5%)	96(22.3%)	129(30.0%)
	> 3 persons	87(16.8%)	20(23.0%)	25(28.7%)	19(21.8%)	32(36.8%)
COVID-19 experienced						
	Never at risk	173(33.5%)	35(20.2%)	39(22.5%)	35(20.2%)	46(26.6%)
	Not sure	149(28.8%)	37(24.8%)	47(31.5%)	33(22.1%)	47(31.5%)
	Experienced risk (ever screening test)	179(34.6%)	42(23.5%)	50(27.9%)	43(24.0%)	62(34.6%)
	Have been infected	16(3.1%)	3(18.8%)	3(18.8%)	4(25.0%)	6(37.5%)

The Copenhegen Burnout Inventory (CBI) is an instrument to measure occupational burnout with excellent psychometric properties and is available in the public domain. The remaining three sections were dedicated to CBI items (Table 2), including: 1) personal-related burnout (5 items), 2) work-related burnout (6 items), and 3) client-related burnout (8 items). Each item was rated on a scale of never or almost never (0), a few times a month (1), once or twice a week (2), three to five times a week (3), and almost every day (4).

**Table 2:** Frequency of personal burnout items.

Questions burnout items		Never or almost never	A few times a month	Once or twice a week	Three to five times a week	Almost every day
**Personal related burnout**		*n* (%)				
1	Feeling tired	52 (10.1%)	189 (36.6%)	152 (29.4%)	83 (16.1%)	41 (7.9%)
2	Physically exhausted	73 (14.1%)	179 (34.6%)	141 (27.3%)	89 (17.2%	35 (6.8%)
3	Emotionally exhausted	215 (41.6%)	179 (34.6%)	141 (27.3%)	89 (17.2%)	35 (6.8%)
4	Cannot take it anymore	171 (33.1%)	203 (39.3%)	84 (16.2%)	40 (7.7%)	19 (3.7%)
5	Feeling worn out	142 (27.5%)	204 (39.5%)	78 (15.1%)	64 (12.4%)	29 (5.6%)
**Work related burnout**						
6	Feeling weak and susceptible to illness	127 (24.6%)	175 (33.8%)	130 (25.1%)	51 (9.9%)	34 (6.6.)
7	Work emotionally exhausting	187 (36.2%)	175 (38.8%)	91 (17.6%)	46 (8.9%)	18 (3.5%)
8	Feeling burnt out because of work	90 (17.4%)	86 (16.6%)	124 (24.0%)	153 (29.6%)	64 (12.4%)
9	Work frustrates you	139 (26.9%)	199 (38.5%)	93 (18.0%)	59 (11.4%)	27 (5.2%)
10	Feeling worn out at the end of working day	242 (46.8%)	156 (30.2%)	65 (12.6%	36 (7.0%)	18 (35.0%)
11	Exhausted in the morning at the thought of another day at work	185 (35.8%)	195 (37.7%)	66 (12.8%)	51 (9.9%)	20 (3.9%)
**Client related burnout**						
12	Feeling every working hour is tiring	135 (26.1%)	212 (41.0%)	93 (18.0%)	52 (10.1%)	25 (4.8%)
13	Not having enough energy during leisure time	78 (15.1%)	171 (33.1%)	131 (25.3%)	73 (14.1%)	64 (12.4%)
14	Hard to work with clients	72 (13.9%)	114 (22.1%)	136 (26.3%)	127 (24.6%)	68 (13.2%)
15	Frustrating to work with clients	98 (19.0%)	141 (27.3%)	129 (25.0%)	112 (21.7%)	37 (7.2%)
16	Draining energy to work with clients	115 (30.0%)	168 (32.5%)	16 (22.4%)	53 (10.3%)	25 (4.8%)
17	Feeling that you give more than you get back when working with clients	68 (13.2%)	153 (39.6%)	106 (20.5%)	88 (17.0%)	102 (19.7%)
18	Feeling tired of working with clients	124 (24.0%)	175 (33.8%)	104 (20.1%)	70 (13.5%)	44 (8.5%)
19	Wondering how long you will be able to continue working with clients	170 (32.9%)	153 (39.6%)	86 (16.6%)	53 (10.3%)	55 (10.6%)

For this study, we used the Thai translation of the CBI (T-CBI), which was found to have a Cronbach’s alpha coefficient of 0.88 to 0.93 from previous reports [21], but in this study, Cronbach’s alpha coefficient showed 0.91 and content validity 0.67 to 1.00. We calculated the average scores for each dimension, where an average score of 50% or above is treated as burnout [22, 23] (see Table 2 for details).

### Statistical analysis

We summarized the descriptive statistics to examine personal-, work-, and client-related burnout prevalence and frequency of personal burnout items. The comparison between factors contributing and burnout items was used in the independent t-test and one-way analysis of variance (ANOVA). The factor variables associated with burnout among healthcare workers were used in multiple linear regression models. The level of significance was set at p < 0.05. The statistical analysis was performed using the Statistical Package for the Social Sciences Program (SPSS), version 22.

## Results

### Participants’ characteristics

A total of 517 questionnaire responses were obtained; the socio-demographics are presented in Table 1. The majority of the participants were medical staff (55.3%), female (83.4%), older than 35 years (59.4%), education higher than bachelor (76.2%), and income more than 25,000 Thai-Bath/month (50.9%). In addition, the largest group of participants (52.8%) was single, living in a household (52.6%), and had children living in the household 302 (58.4%). Most participants (65.6%) did not have diseases but had family members who had disease (63.6%).

In terms of work experience characteristics, most of the participants (57.3%) were experienced more than 10 years, days off (61.3%) were more than 8 days per month, sleeping for less than six hours per day (61.5%), and the number of colleagues less than 3 persons (83.2%). These results indicate that the population was representative of the wider population of HCWs residing in Bangkok. Most reported that they had never been in a situation in which they risked contracting COVID-19 (33.5%) and experienced risk (ever screening test) (34.6%).

### Burnout prevalence among healthcare workers during the COVID-19 pandemic

The burnout prevalence is presented in Table 1. The prevalence of overall, the highest prevalence of burnout was among medical staff (25.9%). As shown in **Table 1**, there was more burnout prevalence among medical staff in all three dimensions of the CBI: personal-, work-, and client-related (29.4%, 26.2%, and 34.3%, respectively).

The prevalence of personal-related burnout was the highest among medical staff that had an education less than a bachelor’s degree, had children living in home, slept for less than six hours per day, and were not sure if they had experienced COVID-19.

The prevalence of work-related burnout was the highest among medical staff that had less than eight days off per month and had experienced being infected with COVID-19.

The prevalence of client-related burnout was the highest among medical staff that had an education less than a bachelor’s degree, had more than three colleagues, had less than eight days off per month, slept less than six hours per day, and had experienced being infected with COVID-19.

### Frequency of personal burnout items

The frequency of personal burnout items is presented in Table 2. Personal-, work-, and client-related burnout items were the majority with respondents, with a few times a month and a few with three to five times a week.

### Comparison between factor variables contributing to burnout among health care workers

Our study presented a comparison between factor variables contributing and burnout among HCWs in Table 3.

**Table 3:** Comparison between contributing factors and burnout.

Contributing factor variables		Overall burnout		Personal-related		Work-related		Client-related	
		mean ± SD	*p*-value	mean ± SD	*p*-value	mean ± SD	*p*-value	mean ± SD	*p*-value
Occupational									
	Medical staff	28.97 ± 14.07	0.008	7.16 ± 4.76	0.064	8.54 ± 5.05	<0.001	13.26 ± 5.62	0.079
	Support staff	25.65 ± 14.01		6.38 ± 4.75		6.88 ± 5.23		12.38 ± 5.71	
Gender									
	Male	23.90 ± 13.63	0.010	5.97 ± 4.54	0.072	6.56 ± 4.95	0.016	11.36 ± 5.93	0.007
	Female	28.21 ± 14.13		6.98 ± 4.78		8.05 ± 5.21		13.16 ± 5.58	
Age (Years)									
	≤ 35	28.52 ± 13.43	0.169	7.02 ± 4.66	0.421	8.31±4.89	0.064	13.18 ± 5.21	0.294
	> 35	26.78 ± 14.57		6.68 ± 4.82		7.45±5.37		12.65 ± 5.97	
Education									
	< bachelor	26.70 ± 14.38	0.479	6.86 ± 4.71	0.894	7.20 ± 5.12	0.141	12.63 ± 6.17	0.600
	≥ bachelor	27.74 ± 14.06		6.80 ± 4.77		7.99 ± 5.21		12.94 ± 5.51	
Income (Baht)									
	≤ 25,000	26.61 ± 13.42	0.166	6.63 ± 4.72	0.382	7.20 ± 4.75	0.107	12.77 ± 5.52	0.727
	> 25,000	28.34 ± 14.76		7.00 ± 4.79		8.38 ± 5.54		12.95 ± 5.82	
Marital status*									
	Sigle	28.23 ± 14.12	0.388	6.90 ± 4.81	0.860	8.37 ± 5.15	0.030	12.96 ± 5.44	0.404
	Married	26.87 ± 14.11		6.68 ± 4.69		7.23 ± 5.19		12.95 ± 5.96	
	Separated	25.47 ± 14.34		7.00 ± 4.82		6.83 ± 5.21		11.63 ± 5.64	
Children									
	No	28.55 ± 14.07	0.044	7.09 ± 4.83	0.118	8.400 ± 5.14	0.002	13.05 ± 5.44	0.373
	Yes	26.01 ± 14.11		6.43 ± 4.63		6.97 ± 5.17		12.60 ± 5.98	
Diseases									
	No	27.67 ± 13.50	0.689	6.82±4.51	0.985	8.00 ± 5.02	0.223	12.84 ± 5.45	0.891
	Yes	27.15 ± 15.28		6.81±5.21		7.42 ± 5.51		12.91 ± 6.09	
Family diseases									
	No	27.24 ± 14.14	0.761	6.46 ± 4.84	0.204	7.72 ± 5.09	0.797	13.04 ± 5.64	0.587
	Yes	27.63 ± 14.14		7.02±4.70		7.85 ± 5.26		12.76 ± 5.69	
Residence*									
	Home	27.25 ± 14.50	0.070	6.88 ± 4.89	0.224	7.63 ± 5.36	0.005	12.73 ± 5.79	0.356
	Condominium	26.53 ± 13.06		6.43 ± 4.40		7.36 ± 4.90		12.73 ± 5.43	
	Hospital	31.16 ± 15.02		7.60 ± 5.07		9.75 ± 4.95		13.81 ± 5.80	
Work experience (Years)									
	≤ 10	28.61 ± 13.72	0.120	7.04 ± 4.76	0.364	8.36 ± 5.00	0.036	13.21 ± 5.41	0.234
	> 10	26.65 ± 14.40		6.65 ± 4.75		7.39 ± 5.31		12.61 ± 5.86	
Days off (per month)									
	< 8	28.74 ± 14.43	0.112	7.20 ± 4.78	0.144	8.46 ± 5.22	0.023	13.07 ± 5.81	0.512
	≥ 8	26.70 ± 13.90		6.57 ± 4.73		7.39 ± 5.15		12.73 ± 5.57	
Sleep (hours per day)									
	≤ 6	29.08 ± 14.02	0.001	7.34 ± 4.73	0.002	8.50 ± 5.07	<0.001	13.23 ± 5.55	0.061
	> 6	24.95 ± 13.97		5.98 ± 4.68		6.69 ± 5.22		12.27 ± 5.82	
Number of colleagues									
	≤ 3 persons	27.31 ± 14.13	0.523	6.77 ± 4.71	0.610	7.71 ± 5.26	0.357	12.83 ± 5.71	0.749
	> 3 persons	28.37 ± 14.14		7.05±4.98		8.27±4.85		13.04±5.52	
COVID-19 experience*									
	Never at risk	25.93 ± 14.72	0.097	6.29 ± 5.07	0.276	7.26 ± 5.37	0.031	12.36 ± 5.67	0.222
	Not sure	27.18 ± 13.57		6.95 ± 4.63		7.41 ± 5.16		12.81 ± 5.73	
	Experienced risk (ever screening test)	29.49 ± 13.89		7.25 ± 4.53		8.73 ± 4.96		13.50 ± 5.63	
	Have been infected	24.93 ± 13.90		6.37 ± 4.77		6.93 ± 5.27		11.62 ± 5.28	

Data were analyzed using the independent t-test and one way analysis of variance (ANOVA)*

In terms of the overall burnout score, the majority of medical staff (28.97 ± 14.07) showed a significant burnout score (p-value 0.008). In addition, females (28.21 ± 14.13), with no children (28.55 ± 14.07), and with less than six hours of sleep per day (29.08 ± 14.02) showed a significant burnout score (p-value < 0.05).

In terms of the personal-related burnout score, our study showed that medical staff (7.16 ± 4.76) and females (6.98 ± 4.78) were significantly likely to have burnout (p-value 0.068). In addition, the majority slept less than six hours per day (7.34 ± 4.73) and showed a significant burnout score (p-value 0.002).

In terms of the work-related burnout score, the majority of the medical staff (8.54 ± 5.05) and females (8.05 ± 5.21) showed a significant burnout score (p-value < 0.001). The participants who were single (8.37 ± 5.15), had no children (8.400 ± 5.14), showed a significant burnout score (p-value < 0.001). In addition, those living in hospital (9.75 ± 4.95), had work experience of less than 10 years (8.36 ± 5.00), and slept less than six hours per day (8.50 ± 5.07) showed a significant burnout score (p-value < 0.001). Moreover, those who had experienced risk (ever screening test) with COVID-19 (8.73 ± 4.96) showed a significant burnout score (p-value < 0.001).

In terms of the client-related burnout scores, our study showed that medical staff (13.26 ± 5.62) who slept less than six hours per day (13.23 ± 5.55) were likely to have a significant burnout score (p-value 0.07). In addition, females (11.36 ± 5.93) had a significant burnout score (p-value 0.007).

### Association between factor variables and burnout among healthcare workers

Table 4 shows the association of the factor variables burnout score among HCWs during the COVID-19 pandemic based on multiple linear regression models.

**Table 4:** Factor variables associated with burnout among healthcare workers.

	Factor variable	Overall burnout		Personal-related		Work-related		Client-related	
		β (95% CI)	*p*-value	β (95% CI)	*p*-value	β (95% CI)	*p-*value	β (95% CI)	*p*-value
Occupational									
	Medical staff	Ref.							
	Support staff	-0.074 (-4.684, 0.463)	0.108	-0.040 (-1.259, 0.488)	0.386	-0.109 (-2.078, -0.210)	0.016	-0.051 (-1.626, 0.464)	0.275
Gender									
	Male	Ref.							
	Female	0.088 (0.033, 6.614)	0.048	0.060 (-0.354, 1.879	0.180	0.070 (-0.218, 2.170)	0.109	0.104 (0.248, 2.921)	0.020
Age (Years)									
	≤ 35	Ref.							
	> 35	0.023 (-3.290, 4.619)	0.741	0.022 (-1.126, 1.558)	0.752	0.037 (-1.045, 1.825)	0.594	0.005 (-1.548, 1.665)	0.943
Children									
	No	Ref.							
	Yes	-0.055 (-4.259, 1.126)	0.254	-0.053 (-1.421, 0.406)	0.276	-0.095 (-1.979, -0.024)	0.045	-0.005 (-1.151, 1.036)	0.918
Work experience (Years)									
	≤ 10	Ref.							
	> 10	-0.070 (-5.926, 1.911)	0.315	-0.039 (-1.709, 0.950)	0.576	-0.092 (-2.390, 0.453)	0.181	-0.058 (-2.252, 0.932)	0.416
Days off (days per month)									
	< 8	Ref.							
	≥ 8	-0.041 (-3.724, 1.356)	0.360	-0.042 (-1.273, 0.450)	0.349	-0.061 (-1.569, 0.273)	0.168	-0.011 (-1.156, 0.907)	0.813
Sleep (hours per day)									
	≤ 6	Ref.							
	> 6	-0.120 (-6.012, -0.969)	0.007	-0.124 (-2.068, -0.357)	0.006	-0.141 (-2.419, -0.589)	0.001	-0.066 (-1.798, 0.250)	0.138

Data were analyzed using the multiple linear regression models. Data were presented as β coefficients and 95% confidence interval (CI).

Female (vs. Male, β 0.088; 95% CI 0.033, 6.614) was higher associated with the overall burnout score. In addition, sleeping for more than six hours per day (vs. ≤6, β -0.120; 95% CI -6.012, -0.969) was lower associated with burnout.

In terms of personal-related burnout, sleeping for more than six hours per day (vs. ≤6, β -0.124; 95% CI -2.068, -0.357) was lower associated with burnout.

In terms of work-related burnout, support staff (vs. medical staff, β -0.109; 95% CI -2.078, -0.210) who slept more than six hours per day (vs. ≤6 hr./day, β -0.141; 95% CI -2.419, -0.589) were lower associated with burnout.

In terms of client-related burnout, female (vs. Male, β 0.104; 95%CI 0.248, 2.921) was higher associated with burnout.

## Discussion

In the current situation, the COVID-19 pandemic has been a source of risk for burnout for both individuals and communities. This is the first urban community in Bangkok, Thailand (n = 517) on burnout among HCWs, based on CBI, conducted during the COVID-19 pandemic. To the best of our knowledge, only a few studies focused on HCWs have been interested in the impact of COVID-19 through a cross-sectional survey. The study found that HCWs as medical staff have a higher prevalence of burnout prevalence than support staff, which indicates that medical staff undergo extreme psychological burnout and are concerned about their future careers, and based on the real COVID-19 situation, the prevalence was lower than the prevalence obtained from Malaysia (53.8%), Singapore (49.2%), and India (44.6%) [23–25]. These findings were similar to studies done in China (13–39%) and Japan (31.4%) [26, 27]. It is difficult to compare our finding with previous literature, as most studies used different scales. Burnout among medical staff has been shown to have a detrimental effect during previous pandemics, revealed by significantly high distress levels among nursing staff, doctors, and healthcare assistants, in a decreasing trend in that order [24, 28]. The COVID-19 outbreak, with its rapid global spread, possibly worsened burnout, as it presented unprecedented challenges to HCWs.

This burnout study in the COVID-19 era using the CBI scale showed personal-, work-, and client-related burnout prevalence among medical staff (29.4%), (26.2%), and (34.3%), respectively. Hence, we found the prevalence of client-related burnout (pandemic-related) to be the highest in all categories of HCWs. Therefore, the overall burnout and burnout in the three domains were mainly related to job insecurity, high workload, and lack of job satisfaction, and were higher [29] and lower [30] than in previous studies. Our study suggested that burnout prevalence might come from feeling burnt out because of work, hard to work with clients, and feeling tired of working with clients during the COVID-19 pandemic. In addition, the predictors of burnout were a lack of interest in the job, insecurity, history of physical illness, poor relationship with superiors, and worry about getting infected or falling ill. Moreover, the burnout among paramedics was probably due to long working hours, job insecurity, being undervalued as a workforce, poor remuneration, and the lack of support from superiors [24]. Our study showed that participants (34.6%) feared the risk of experiencing COVID-19 during working hours. Other studies have also shown that the history of contact with the patient was an independent risk factor [31].

Our study found that females were more likely to experience burnout prevalence than males, indicating that in men, depersonalization and personal accomplishment are only associated with depression (although this relationship is not significant), while in women it is associated with the three scales of burnout [32]. In addition, the chances of having severe symptoms increased if the respondents were females, had intermediate seniority, and worked on the frontlines [24]. One study suggested that the risk factors for depression in women are likely to be of biological origin, such as fluctuations in hormone levels, as seen during the changes in the menstrual cycle [33]. This may be because during the pandemic, compared with power factors, such as infection control, the influence of the gender variable and age variable was masked [34].

Our study presented that sleeping for less than six hours per day might be one factor associated with burnout prevalence that may be slightly low. This evidence may come from real satiation based on workloads. There were 61.5% of the participants who had less than six hours of sleep per day. This might be a trend of burnout in many areas [35]. Some studies have shown that the risks of sleep deprivation were noticeable among HCWs during the pandemic [34]. This practice gave HCWs some time to rest and reduced HCWs’ concerns that they would pass the virus to their families and friends.

In terms of socio-demographics, age, having children in the household, work experience, and days off were not associated with burnout prevalence. Although it was not described in this study, we found that inadequate support for HCWs during COVID-19 increased the odds of developing burnout. In addition, some studies have suggested that burnout may develop because of other factors such as dissatisfaction with the job, high workload, not feeling appreciated by senior management, inadequate remuneration, failure to achieve goals, poor interpersonal relationships at the workplace, competing family interests with lack of time with family, which can all contribute to the development of burnout [24].

This study highlights the importance of addressing burnout among HCWs, with medical staff, females, and sleeping less than six hours per day being associated with burnout. Our study would suggest further that both identification and interventions are needed for frontline HCWs to prevent and reduce the risk of burnout, with a high proportion of females compared to males. Thus, the government should provide support in these areas to prevent a humanitarian crisis.

Our study has a few limitations. First, the data are cross-sectional, and a causal relationship could not be confirmed. Second, the data were collected from participants’ self-reports; thus, inherent bias was unavoidable. Third, the survey did not cover all the related factors for burnout in health professionals. Finally, we designed the questionnaire according to the practical situation of an urban community in Bangkok, Thailand, and due to the period, we designed the questionnaire, literature related to burnout of HCWs among the COVID-19 pandemic was lacking. Perhaps there were some factors that affected the results that we did not include in our study.

## Conclusions

During the COVID-19 pandemic, factors such as HCWs, age, gender, hours slept, having children in the household, work experience, and days off. HCWs who were medical staff, female and hours slept were the majority on burnout prevalence in Bangkok, Thailand. Contributing factors were also feeling burnt out because of work, hard to work with clients and feel tired of working with clients during COVID-19 pandemic. However, COVID-19 has been ongoing for a year, and the continuous strain may exhaust HCW resources from coping and burnout. This study further explores the interventions for frontline HCWs to prevent and reduce the risk of burnout that are needed among the high proportion of females compared to males.

## Supporting information

S1 File The Institutional Review Board of the Faculty of Medicine Vajira Hospital is in full compliance with the international guidelines for human research protection as Declaration of Helsinki, The Belmont Report, CIOMS Guideline and International Conference on Harmonization in Good Clinical Practice (ICH-GCP). (DOCX)

S2 File The full English language version of the questionnaire. The full English language version of the questionnaire contained all the details of the original Thai version of the questionnaire.
(DOCX)

## Abbreviations

COVID-19: Coronavirus disease
HCWs: Health care workers
CBI: Copenhegen Burnout Inventory
CI: Confidence Interval
