# Supplementary material for: Burnout prevalence and contributing factors among healthcare workers during the COVID-19 pandemic: A cross-sectional survey study in an urban community in Thailand
Source: PLoS One. 2022 Aug 4;17(8):e0269421. doi: 10.1371/journal.pone.0269421 (PMC9352097; doi:10.1371/journal.pone.0269421)

**Additional file 1**

The Institutional Review Board of the Faculty of Medicine Vajira Hospital is in full compliance with the international guidelines for human research protection as Declaration of Helsinki, The Belmont Report, CIOMS Guideline and International Conference on Harmonization in Good Clinical Practice (ICH-GCP).


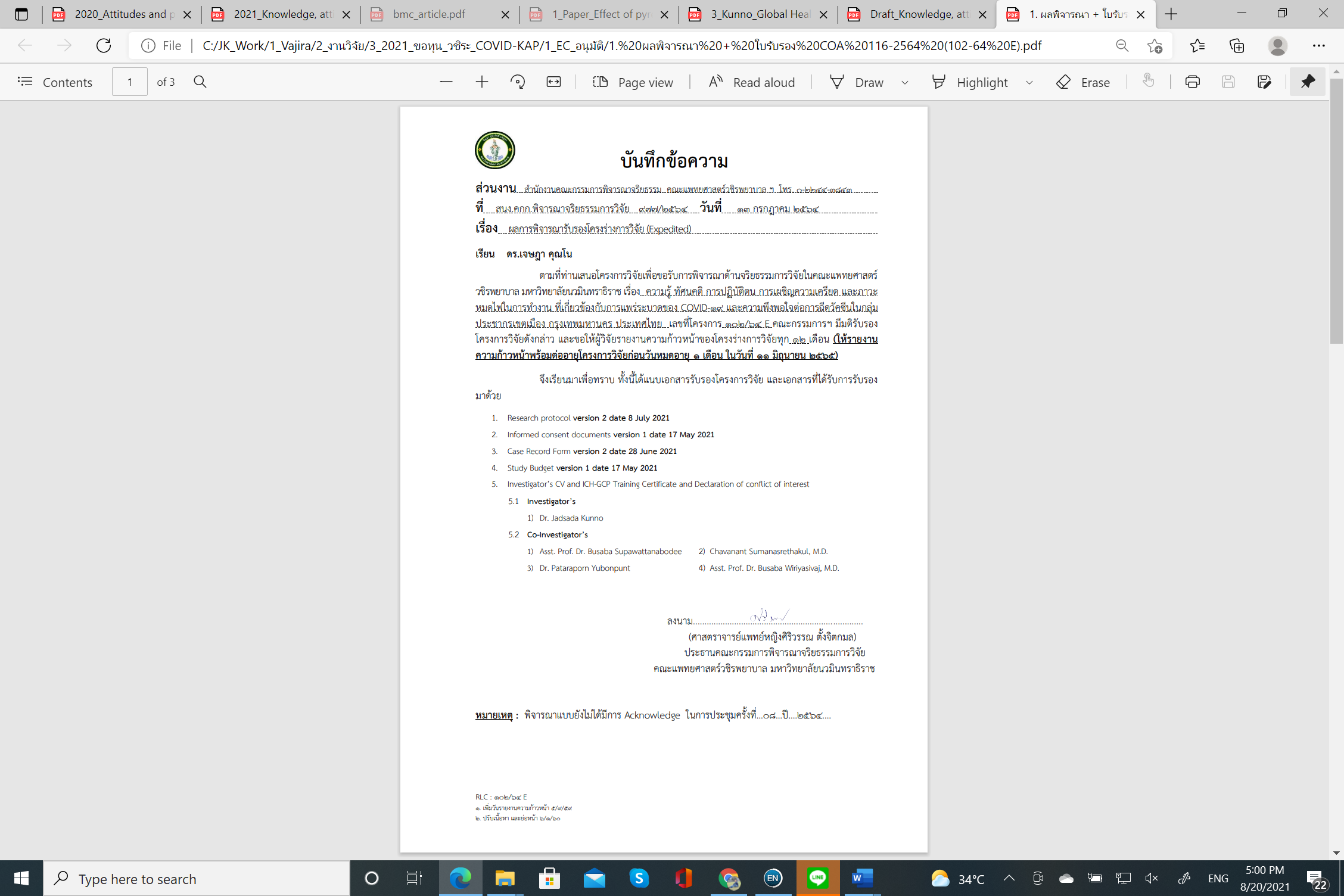


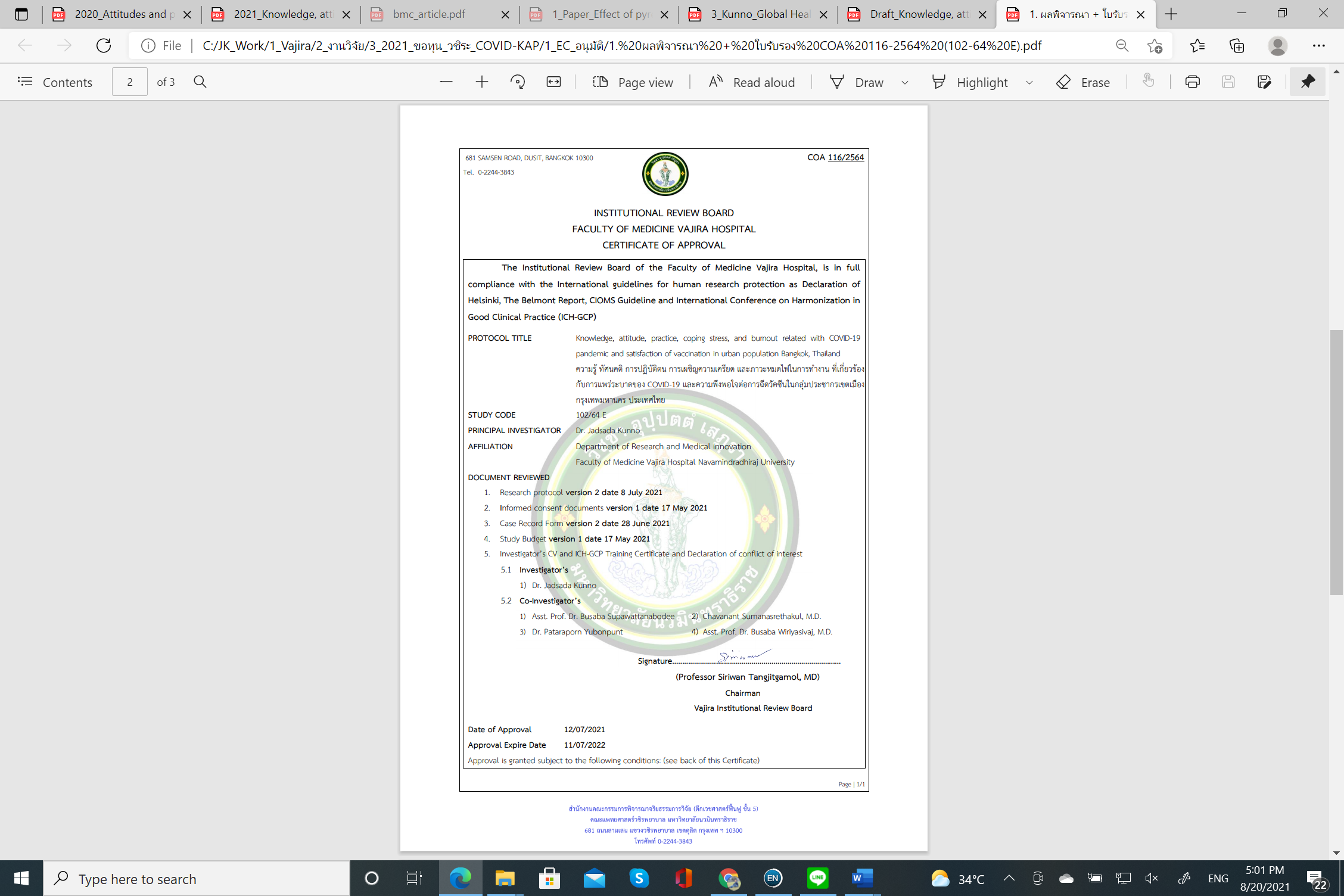


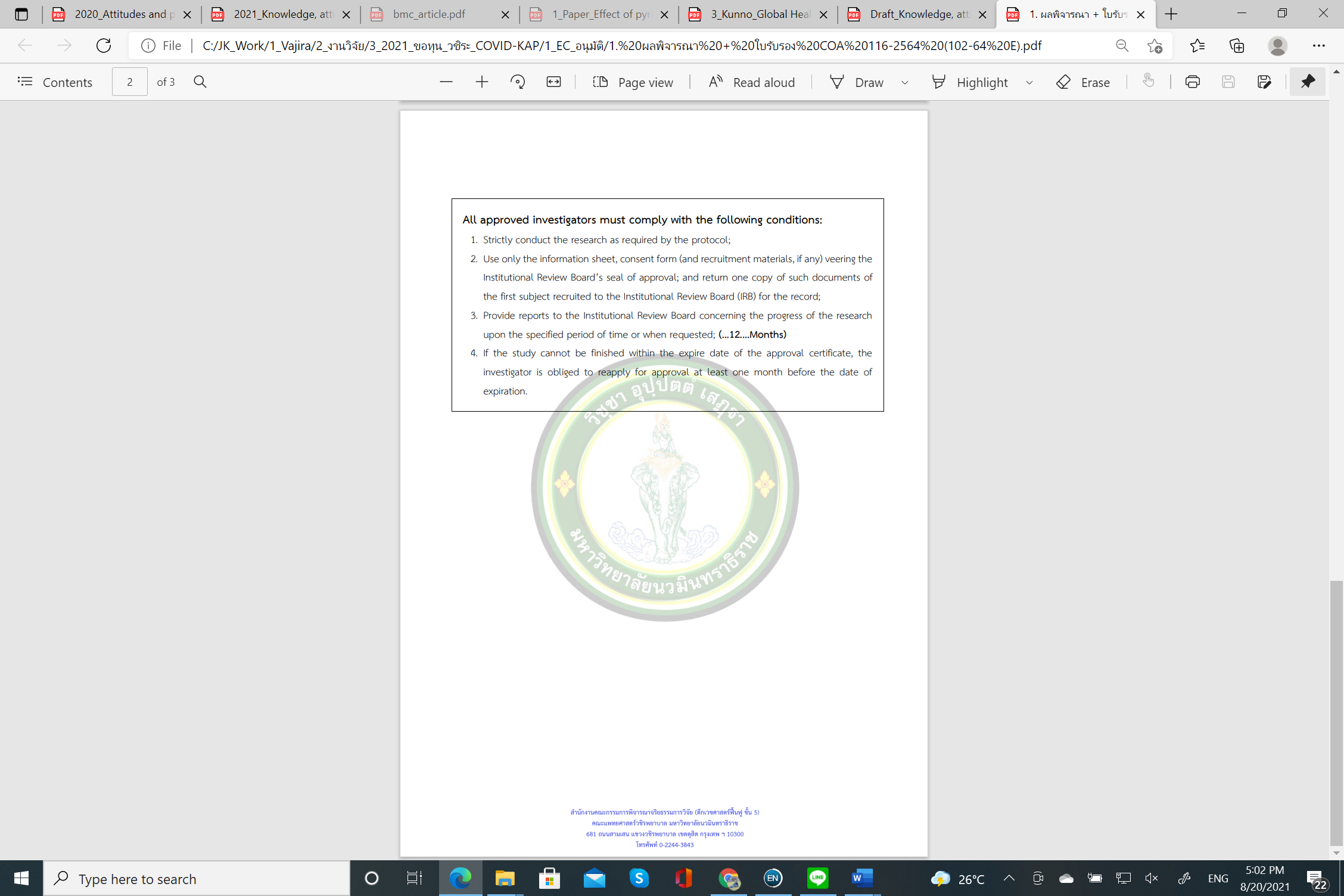


This study was approved by the ethics committee of Faculty of Public and Environmental Health, Huachiew Chalermprakiet University, Samutprakan, Thailand, (อ.1102/2564 ลว).


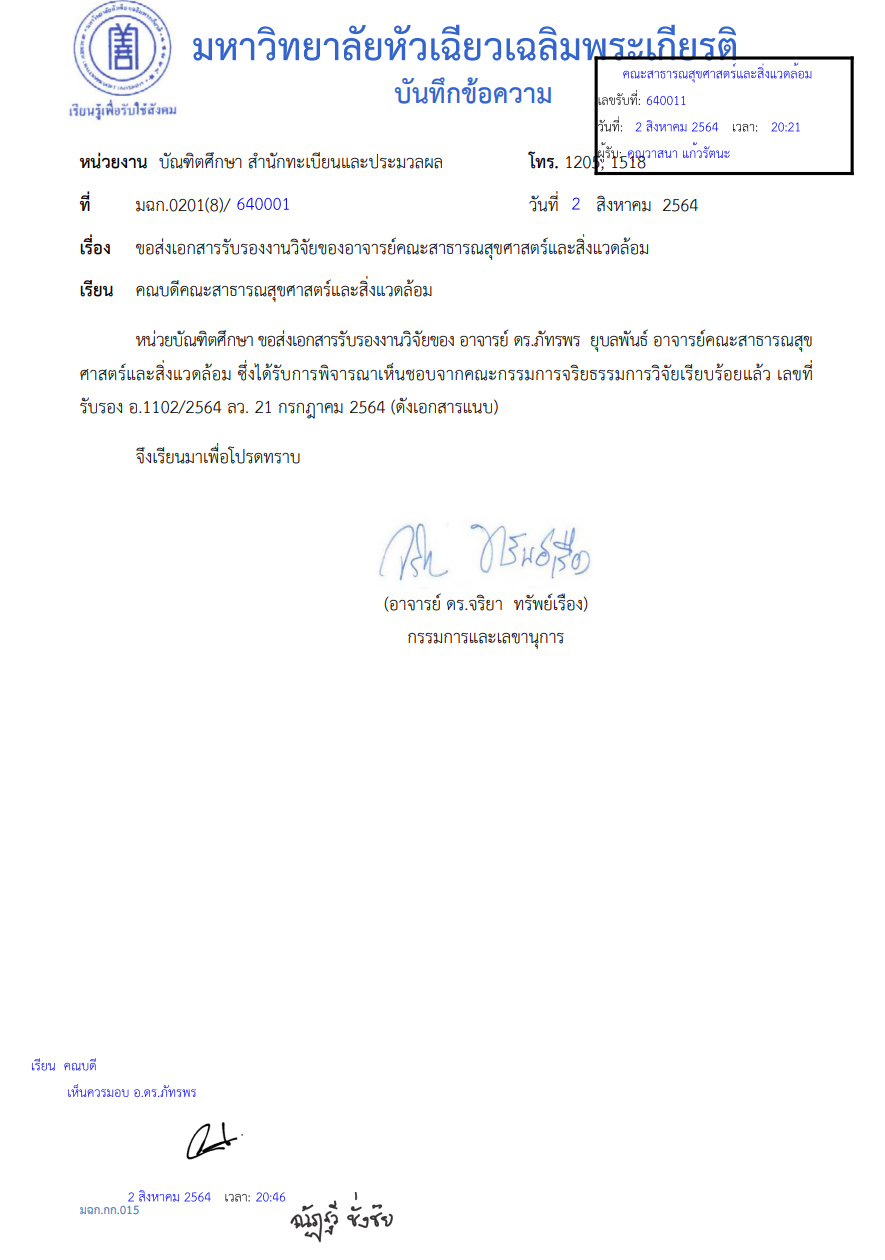


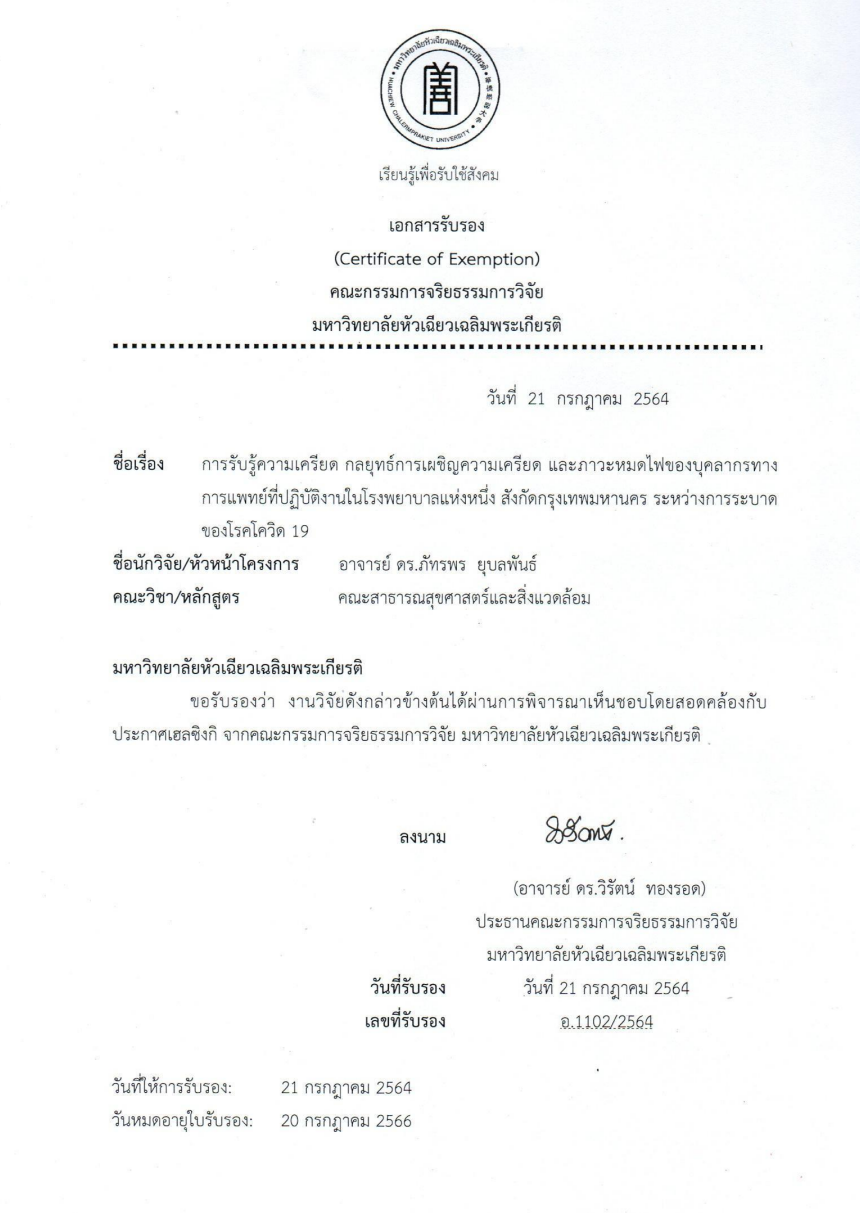

Supplement: S1 File — (DOCX) [file pone.0269421.s001.docx]
