# Supplementary material for: Burnout prevalence and contributing factors among healthcare workers during the COVID-19 pandemic: A cross-sectional survey study in an urban community in Thailand
Source: PLoS One. 2022 Aug 4;17(8):e0269421. doi: 10.1371/journal.pone.0269421 (PMC9352097; doi:10.1371/journal.pone.0269421)
Supplement: S2 File — The full English language version of the questionnaire contained all the details of the original Thai version of the questionnaire. (DOCX) [file pone.0269421.s002.docx]

**Additional file 2**

The full English language version of the questionnaire. The full English language version of the questionnaire

**Questionnaire (English version)**

**Consent from**

**( ) Allow ( ) Not allow**

**ID ………………**

**The questionnaire is divided into four sections.**

**Section 1.**

1. Occupational

( ) Medical staff ( ) Support staff

1. Gender

( ) Male ( ) Female

1. Age (Years)

( ) ≤ 35 ( ) > 35

1. Education

( ) < bachelor ( ) ≥ bachelor

1. Income (Baht)

( ) ≤ 25,000 ( ) > 25,000

1. Marital status*

( ) Sigle ( ) Married ( ) Separated

1. Children

( ) No ( ) Yes

1. Diseases

( ) No ( ) Yes

1. Family diseases

( ) No ( ) Yes

1. Residence*

( ) Home ( ) Condominium ( ) Hospital

1. Work experience (Years)

( ) ≤ 10 ( ) > 10

1. Days off (per month)

( ) < 8 ( ) ≥ 8

1. Sleep (hours per day)

( ) ≤ 6 ( ) > 6

1. Number of colleagues

( ) ≤ 3 persons ( ) > 3 persons

1. COVID-19 experienced

( ) Never at risk ( ) Not sure ( ) Experienced risk (ever screening test) ( ) Have been infected

**Section 2.**

Burnout items

| **Questions burnout items** | | **Never or almost never** | **A few times a month** | **Once or twice a week** | **Three to five times a week** | **Almost every day** |
| --- | --- | --- | --- | --- | --- | --- |
| **Personal related burnout** | | *n* (%) | | | | |
| 1 | Feeling tired |  |  |  |  |  |
| 2 | Physically exhausted |  |  |  |  |  |
| 3 | Emotionally exhausted |  |  |  |  |  |
| 4 | Cannot take it anymore |  |  |  |  |  |
| 5 | Feeling worn out |  |  |  |  |  |
| **Work related burnout** | |  |  |  |  |  |
| 6 | Feeling weak and susceptible to illness |  |  |  |  |  |
| 7 | Work emotionally exhausting |  |  |  |  |  |
| 8 | Feeling burnt out because of work |  |  |  |  |  |
| 9 | Work frustrates you |  |  |  |  |  |
| 10 | Feeling worn out at the end of working day |  |  |  |  |  |
| 11 | Exhausted in the morning at the thought of another day at work |  |  |  |  |  |
| **Client related burnout** | |  |  |  |  |  |
| 12 | Feeling every working hour is tiring |  |  |  |  |  |
| 13 | Not having enough energy during leisure time |  |  |  |  |  |
| 14 | Hard to work with clients |  |  |  |  |  |
| 15 | Frustrating to work with clients |  |  |  |  |  |
| 16 | Draining energy to work with clients |  |  |  |  |  |
| 17 | Feeling that you give more than you get back when working with clients |  |  |  |  |  |
| 18 | Feeling tired of working with clients |  |  |  |  |  |
| 19 | Wondering how long you will be able to continue working with clients |  |  |  |  |  |
